# First Case of Respiratory Infection in Rabbits Caused by *Alcaligenes faecalis* in Romania

**DOI:** 10.3390/vetsci12010033

**Published:** 2025-01-09

**Authors:** Vlad Iorgoni, Ionica Iancu, Ionela Popa, Alexandru Gligor, Gabriel Orghici, Bogdan Sicoe, Corina Badea, Cristian Dreghiciu, Călin Pop, Timea Bochiș, Janos Degi, Luminita Costinar, Corina Pascu, Viorel Herman

**Affiliations:** 1 Department of Infectious Diseases and Preventive Medicine, Faculty of Veterinary Medicine, University of Life Sciences “King Mihai I”, 300645 Timișoara, Romania; vlad.iorgoni@usvt.ro (V.I.); alexandru.gligor@usvt.ro (A.G.); corina.badea@usvt.ro (C.B.); janosdegi@usvt.ro (J.D.); luminita.costinar@usvt.ro (L.C.); corinapascu@usvt.ro (C.P.); viorel.herman@fmvt.ro (V.H.); 2 Department of Microbiology, Faculty of Veterinary Medicine, University of Life Sciences “King Mihai I”, 300645 Timișoara, Romania; ionela.popa@usvt.ro; 3 Department of Veterinary Emergencies, Faculty of Veterinary Medicine, University of Life Sciences “King Mihai I”, 300645 Timișoara, Romania; gabriel.orghici@usvt.ro; 4 Department of Radiology and Imaging, Faculty of Veterinary Medicine, University of Life Sciences “King Mihai I”, 300645 Timișoara, Romania; bogdan.sicoe@usvt.ro; 5 Department of Parasitology, University of Life Sciences “King Mihai I”, 300645 Timișoara, Romania; cristian.dreghiciu@usvt.ro; 6 Department of Semiology, Faculty of Veterinary Medicine, University of Life Sciences “King Mihai I”, 300645 Timișoara, Romania; calinpop@usvt.ro (C.P.); timea.bochis@usvt.ro (T.B.)

**Keywords:** *Alcaligenes faecalis*, rabbit, generalized infection, zoonosis, antibiotic resistance

## Abstract

*Alcaligenes faecalis* is a Gram-negative, obligate aerobic bacillus commonly found in environmental settings, particularly in soil and water, as well as within the intestinal microbiota of humans and various animal species. This bacterium has the potential to act as an opportunistic pathogen, capable of causing severe infections in both humans and animals, which can sometimes result in fatal outcomes. Notably, *A. faecalis* is often characterized by a high degree of antibiotic resistance, complicating treatment efforts. In the current study, we present a case involving a 3-month-old male rabbit that developed a generalized infection, ultimately leading to the animal’s death. Despite the administration of broad-spectrum systemic antibiotics, the treatment was ineffective in salvaging the animal’s health. The isolated strain of *A. faecalis* demonstrated resistance to all tested antibiotics, underscoring the challenges associated with managing infections caused by this pathogen. This case highlights the significant impact of *A. faecalis* as an emerging threat in veterinary medicine, where its inherent antibiotic resistance further exacerbates the difficulty of effective treatment.

## Simple Summary

*Alcaligenes faecalis*, a Gram-negative, aerobic bacterium commonly found in environmental settings and the human intestinal flora, can act as an opportunistic pathogen, particularly in immunocompromised individuals. This study presents a case involving a 3-month-old male German giant rabbit that succumbed to a severe, generalized infection caused by *A. faecalis*. The infection led to a severe infection and eventual death despite broad-spectrum antibiotic treatment. The isolated strain of *A. faecalis* demonstrated resistance to all tested antibiotics, complicating treatment efforts. This case underscores the significant challenges in managing *A. faecalis* infections due to their inherent antibiotic resistance and highlights the need for prompt identification and tailored therapeutic strategies. The findings emphasize the importance of continued surveillance and research into effective antimicrobial treatments for such multidrug-resistant pathogens.

## 1. Introduction

*Alcaligenes faecalis* is a Gram-negative, rod-shaped, nonfermentative, aerobic bacterium belonging to the family *Alcaligenaceae*. It is oxidase-positive, catalase-positive, and nonencapsulated, with a characteristic ability to produce an alkaline reaction in specific media. *A. faecalis* is widely distributed in the environment, particularly in soil and water, and is considered a normal component of the human intestinal microbiota. However, it has also been isolated from various clinical specimens, including urine, blood, wounds, feces, cerebrospinal fluid (CSF), and respiratory secretions [1,2,3].

In hospital settings, *A. faecalis* is frequently found in respirators, hemodialysis systems, and intravenous solutions, often acting as an opportunistic pathogen. This microorganism is predominantly transmitted through contaminated hospital equipment, such as ventilation devices and nebulizers, or via direct contact with infected fluids or surfaces [4,5]. Despite its classification as an opportunistic pathogen, infections caused by *A. faecalis* are relatively rare but can lead to severe outcomes, especially in immunocompromised individuals [6]. Reported infections associated with this bacterium include bacteremia, meningitis, endophthalmitis, otitis media, endocarditis, pneumonia, peritonitis, and urinary tract infections, among others [7,8,9,10]. There are also sporadic cases documented in immunocompetent hosts, although such occurrences are extremely rare [7].

In terms of antimicrobial susceptibility, *A. faecalis* is generally resistant to aminoglycosides and tetracyclines, while remaining susceptible to trimethoprim/sulfamethoxazole (TMP-SMX) and certain β-lactam antibiotics, such as ureidopenicillins, ticarcillin-clavulanic acid, cephalosporins, and carbapenems [11]. However, recent studies have identified genes conferring intrinsic resistance to several β-lactam antibiotics, raising concerns over emerging multidrug-resistant (MDR) and pan-drug-resistant (PDR) strains [12,13]. To date, cases of PDR in *A. faecalis* are exceedingly rare, but the potential for resistance highlights the importance of continued surveillance and appropriate antimicrobial stewardship [14,15,16].

This study presents a case of a 3-month-old rabbit suffering from a generalized infection caused by *A. faecalis*. Despite attempts at treatment with broad-spectrum antibiotics, the therapeutic approach was inadequate, leading to the animal’s death. This case highlights the challenges associated with treating infections caused by *A. faecalis*, particularly in the context of its potential antibiotic resistance.

## 2. Case Study

This study presents the case of a 3-month-old male German giant rabbit (*Oryctolagus cuniculus*) bred and raised on a small-scale household farm in western Romania. The rabbit was part of a breeding program managed by a local breeder who rears rabbits for both exhibition purposes and familial consumption. The rabbits were housed in a shelter lacking adequate ventilation, leading to the accumulation of moisture and ammonia. These suboptimal environmental conditions, exacerbated during the cold season due to the use of a heating system to minimize losses during parturition and early postnatal days, are known to compromise immune function in rabbits.

At approximately two months of age, shortly after weaning, the rabbit exhibited clinical signs including sneezing, lethargy, reduced activity, and a noticeable decline in weight gain. The owner initiated oral treatment with enrofloxacin 10% without veterinary consultation or prior antibiogram testing, based on his prior experience and having access to leftover medication from a previous prescription. The rabbit’s condition temporarily improved during treatment; however, the symptoms reappeared upon cessation, and the animal’s health further deteriorated. A second course of enrofloxacin was administered but failed to produce beneficial effects, ultimately resulting in the rabbit’s death.

**Figure 1:**
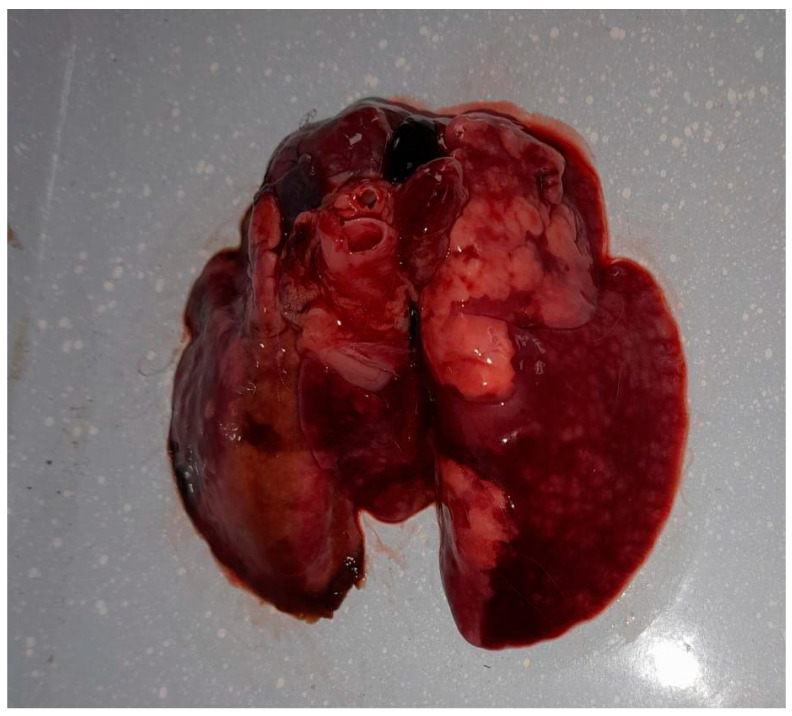
Gross lesions in the lungs of the rabbit infected with *Alcaligenes faecalis*, showing multifocal areas of consolidation, dark red discoloration, and scattered nodular lesions consistent with hemorrhage, edema, and necrosis.

Following the death, the owner sought diagnostic services at the Faculty of Veterinary Medicine in Timișoara to ascertain the cause of death. The preliminary examination revealed a cachectic state and nasal discharge. The animal was already in a precarious condition before the onset of clinical signs, due to suboptimal management practices, and following the appearance of clinical signs associated with the respiratory infection, the animal’s condition deteriorated further, leading to a marked progression of cachexia. A complete necropsy revealed multiple organ lesions, including bronchopneumonia, pulmonary congestion (Figure 1), hydropericardium, cardiac petechiae, an enlarged liver with necrotic areas, and a congested, enlarged spleen. These findings indicated a severe, systemic infection.

To identify the etiological agent, *A. faecalis*, samples were collected from lung tissue and bone marrow from the long bones; no other organs were included in the analysis. These samples were cultured under carefully controlled conditions, including appropriate temperature, nutrient media, and aeration, to ensure optimal growth and accurate isolation of the bacterial strains being investigated.

The primary cultures were conducted on 5% defibrinated sheep blood agar, nutrient agar (Blood Agar Base—Biolab, Hungary), and MacConkey agar, which are standard media for isolating and identifying both Gram-positive and Gram-negative bacteria. The bacterial growth was observed in pure culture, indicating the presence of a single microorganism (*A. faecalis*). No mixed flora or other organisms were detected in the cultures obtained.

**Figure 2:**
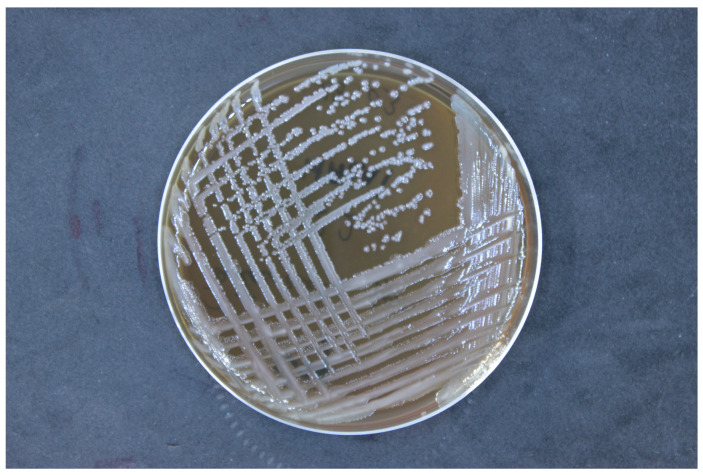
Colony morphology of *Alcaligenes faecalis* on trypticase soy agar, showing smooth, round, convex colonies with a creamy white appearance, characteristic of this bacterial species.

Then, the strain was cultured on nutrient-rich media, trypticase soy agar (TSA). Incubation was conducted at a temperature of 37 °C under aerobic conditions. The pH of the culture medium was maintained within the range of 7.0 to 7.5, and the incubation period was 24 h (Figure 2). The isolated strains were identified using matrix-assisted laser desorption/ionization time-of-flight mass spectrometry (MALDI-TOF MS, Bruker Daltonik, Bremen, Germany). The bacterial samples were prepared using an ethanol/formic acid protocol, applying 1 μL of bacterial protein suspension to an MALDI target plate, followed by the addition of 1 μL of a matrix solution (10 mg α-cyano-4-hydroxycinnamic acid per mL in 50% acetonitrile and 2.5% trifluoroacetic acid). Mass spectra were obtained using the Microflex™ mass spectrometer (Bruker Daltonik) and analyzed with MALDI BioTyper™ 3.0 software, according to the manufacturer’s criteria: a score of ≥2.0 confirmed species-level identification, while scores between 1.7 and 2.0 suggested genus-level identification.

Quality control procedures were rigorously followed to ensure accuracy and reliability of the susceptibility testing. For this purpose, the ATCC 8750 strain, *A. faecalis* strain, was used as a standard reference strain to validate the testing process. The results obtained from the quality control strain were carefully monitored and evaluated to ensure they fell within the established quality control ranges. This confirmation that the *A. faecalis* ATCC 8750 strain’s susceptibility profile remained within acceptable limits provided assurance that the testing methods were accurate and consistent, thereby validating the results obtained for the experimental strains [17,18].

The MALDI-TOF MS analysis confirmed *Alcaligenes faecalis* as the etiological agent responsible for the infection, with an identity similarity index of 2.12. This bacterium was isolated from both the lung tissue and bone marrow, being the only microbe isolated from both lung and bone marrow, indicating a systemic spread and supporting the conclusion that *A. faecalis* was the primary cause of death. The isolation from lung tissue and bone marrow supports the hypothesis of an infection that leads to septicemia.

To further characterize the isolated strain, an antibiogram was performed using the disk diffusion method on Mueller–Hinton agar, following Clinical and Laboratory Standards Institute (CLSI) guidelines [4,19]. The isolated strain displayed resistance to all tested antibiotics, including enrofloxacin, trimethoprim-sulfamethoxazole, oxytetracycline, neomycin, streptomycin, penicillin G, sulfamethoxazole, amoxicillin, colistin sulfate, gentamicin, florfenicol, lincomycin, and doxycycline. The extensive resistance profile of the isolated strain highlights the significant challenge of treating infections caused by *A. faecalis* in clinical settings, especially in light of limited effective therapeutic options.

## 3. Discussion

*Alcaligenes faecalis* is a ubiquitous bacterium found in soil, water, and human intestinal microbiota, and it is utilized in environmental and pharmaceutical applications due to its ability to degrade organic pollutants and produce key biochemical precursors. Although infections with *A. faecalis* are relatively rare, they can be severe, particularly in immunocompromised individuals, neonates, and children. These infections include bacteremia, peritonitis, ocular infections, and urinary tract infections. Recently, *A. faecalis* has emerged as a significant pathogen in hospitalized patients, with notable resistance to multiple antibiotics, including ciprofloxacin, levofloxacin, aminoglycosides, and monobactams [1,7,11,13].

In veterinary medicine, *A. faecalis* has been identified as a pathogen in various animal species, including companion animals and livestock. It has been associated with respiratory and systemic infections in animals, where it can cause severe septicemia and respiratory disease. The rise in multidrug-resistant strains in both human and veterinary settings highlights the critical need for ongoing surveillance and appropriate antimicrobial management [16,20,21,22,23].

In a 2024 study, *A. faecalis*, a significant environmental pathogen, was investigated for antibiotic resistance linked to its presence in poultry farms. Whole-genome sequencing of soil and fecal samples identified four multidrug-resistant strains, showing resistance to vancomycin, ceftazidime, colistin, and ciprofloxacin. Comparative analysis revealed over 180 resistance genes, including those conferring resistance to amoxicillin, and the Mycobacterial insertion element IS6110, suggesting potential for gene transfer [16].

In a study from North Carolina, *A. faecalis* was found to be prevalent in commercial broilers during the winter months. Forty percent of individual birds aged 35 to 45 days and sixty-two percent of flocks tested positive for the bacterium. The bacterium was frequently the predominant microorganism isolated from turbinates and tracheas. Notably, its prevalence was significantly higher in flocks with respiratory disease (75%) compared to those without clinical signs (29%), suggesting a potential causal role in respiratory pathology [21].

Previous reports have documented respiratory infections caused by *A. faecalis* in avian species, particularly affecting young poultry. These cases highlight the pathogen’s ability to cause significant respiratory disease, often leading to systemic infections in birds raised in crowded or suboptimal environmental conditions [16,24].

In a study from France, bacteriological examinations of 60 intranasal swabs from rabbits identified 14 bacterial species, including *A. faecalis*, with no significant differences between animals with or without respiratory diseases. While young weaned rabbits were more often identified as carriers of *Pasteurella multocida*, the role of *A. faecalis* in rabbit respiratory health remains unclear. This highlights the need for further investigation into its pathogenic potential [25].

In a study on the drug-resistant bacteria of the *Alcaligenes* genus isolated from rabbits with intestinal disorders, *A. faecalis* was identified as a significant pathogen. The findings revealed a considerable prevalence of *A. faecalis* in infected rabbits, suggesting a potential role in gastrointestinal diseases. Notably, the study also highlighted the antibiotic resistance profile of *A. faecalis*, which complicates treatment options for affected rabbits [26].

In a case from 2020, a 66-year-old male with multiple comorbidities developed a se-vere *Alcaligenes faecalis* infection resistant to several antibiotics, including fluoroquin-olones and carbapenems. This case illustrates the difficulty of managing multi-drug-resistant infections, particularly in patients with complex medical histories and prolonged ventilator use. The failure of broad-spectrum antibiotics highlights the need for rapid identification and targeted treatment. Additionally, the presence of cavitary lesions and the observed resistance patterns highlight the importance of considering alternative therapies, such as probiotics, bacteriophages, and natural antimicrobial agents, as adjuvants in the treatment of severe cases involving rare and resistant pathogens like *A. faecalis*. These therapies may complement conventional antibiotic treatment, potentially enhancing clinical outcomes in such challenging cases. [13].

A retrospective review at the University Hospital of Guadalajara over six years identified only five cases of skin and soft tissue infections (SSTIs) caused by *Alcaligenes faecalis*. This Gram-negative rod, rarely implicated in pathological processes,, typically found in soil, water, and human intestinal microbiota, has been linked to nosocomial infections, often affecting immunocompromised patients. Most strains were sensitive to amoxicillin/clavulanic acid but resistant to other antibiotics like cefuroxime and ciprofloxacin. This study underscores the need for vigilance in identifying *A. faecalis* infections, especially in patients with predisposing factors, and highlights the challenges in treating infections due to high antibiotic resistance [1].

This case highlights the clinical challenges posed by *A. faecalis*, particularly its potential for multidrug resistance. The systemic infection in a 3-month-old German giant rabbit, despite broad-spectrum antibiotic therapy, underscores the need for precise diagnostic tools such as MALDI-TOF MS and tailored treatments. From the available literature, we could not identify similar cases of systemic *A. faecalis* infection in rabbits. Previously, *A. faecalis* has been isolated from the nasal and intestinal microbiota of rabbits and associated with enteritis in this species. In birds, however, respiratory and systemic infections caused by this pathogen have been described. Given its high resistance to antibiotics and zoonotic potential, *A. faecalis* should be considered in differential diagnoses, particularly in immunocompromised hosts or environments with suboptimal hygiene. Ongoing surveillance and further research are essential to elucidate resistance mechanisms and develop innovative therapeutic approaches for this pathogen [16,21,23,24,25,26].

## 4. Conclusions

This study highlights the clinical challenges of *A. faecalis*, particularly its multidrug resistance and potential for severe systemic infections. The case of a German giant rabbit underscores the need for precise diagnostics and tailored treatments, as broad-spectrum antibiotics were ineffective. While *A. faecalis* has been linked to enteritis in rabbits, systemic infections remain undocumented. Its resistance and zoonotic potential emphasize the importance of surveillance, research into resistance mechanisms, and alternative therapies.

## Data Availability

The original contributions presented in this study are included in the article. Further inquiries can be directed to the corresponding author.
